# Supplementary material for: An electrically controlled single-molecule spin switch
Source: Nat Commun. 2025 Sep 8;16:8242. doi: 10.1038/s41467-025-63574-0 (PMC12417546; doi:10.1038/s41467-025-63574-0)
Supplement: Supplementary file 1 — Supplementary Information [file 41467_2025_63574_MOESM1_ESM.pdf]

Supplementary Information

An electrically controlled single molecule spin switch

Wantong Huang[1*], Kwan Ho Au-Yeung[1*], Paul Greule[1], Máté Stark[1], Christoph Sürgers[1], Wolfgang Wernsdorfer[1,2], Roberto Robles[3], Nicolas Lorente[3,4], Philip Willke[1,5†]

[1] Physikalisches Institut, Karlsruhe Institute of Technology, Karlsruhe, Germany
[2] Institute for Quantum Materials and Technologies, Karlsruhe, Germany
[3] Centro de Física de Materiales CFM/MPC (CSIC-UPV/EHU), 20018 Donostia-San Sebastián, Spain
[4] Donostia International Physics Center, 20018 Donostia-San Sebastián, Spain
[5] Center for Integrated Quantum Science and Technology (IQST), Karlsruhe Institute of Technology, Karlsruhe, Germany

* These authors contributed equally to this work.
† Corresponding author: philip.willke@kit.edu

Table of Contents

Supplementary Note 1: Line profiles of State A and B complexes 2
Supplementary Note 2: Lattice site analysis 2
Supplementary Note 3: Additional DFT calculations 3
Supplementary Note 4: Reversible switching of State A and B 8
Supplementary Note 5: Additional data of switching different Fe-FePc complexes 9
Supplementary Note 6: Molecular rotation beyond the switching threshold 9
Supplementary Note 7: Additional statistical data for molecular rotation 10
Supplementary Note 8: Z-offset dependent I-V spectroscopy 11
Supplementary Note 9: Setpoint dependent dI/dV spectra using a magnetic tip 12
Supplementary Note 10: Spatially resolved dI/dV across the spin switch in State A 13
Supplementary Note 11: Residual tip magnetic field effect 13
Supplementary Note 12: Resonance frequency shift of FePc target induced by spin switch..14
Supplementary Note 13: Energy level diagram of the State A complex 15
Supplementary Note 14: Lattice fit analysis of State A and B 16

Supplementary Note 1: Line profiles of State A and B complexes

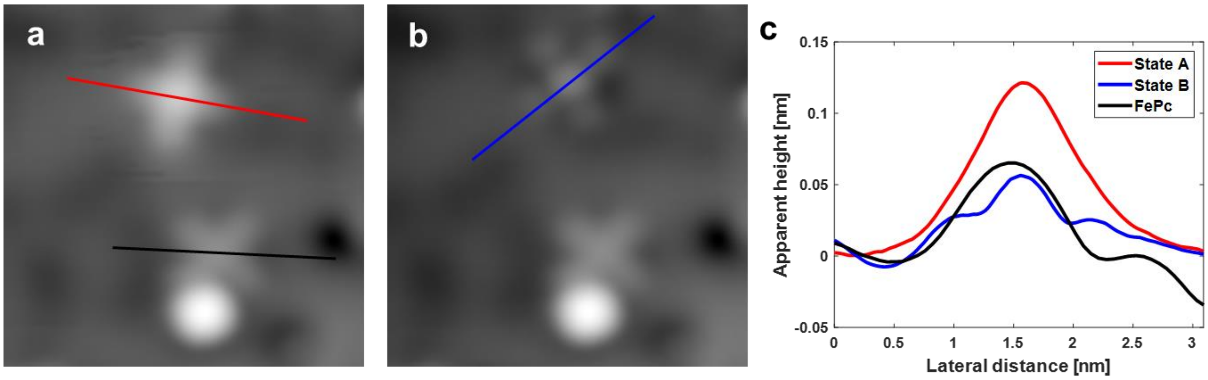

Supplementary Fig. 1. **Line profiles of State A and B complexes.** Comparison of line profiles of (a) State A and (b) State B. The profile of FePc is shown as a reference. The red, blue, and black lines indicate the corresponding plot of apparent height over lateral distance in (c). The apparent height difference between A and B measured at the molecular center is 0.07 nm. STM images: 5 nm x 5 nm; V_{DC} = -100 mV, I = 20 pA.

Supplementary Note 2: Lattice site analysis

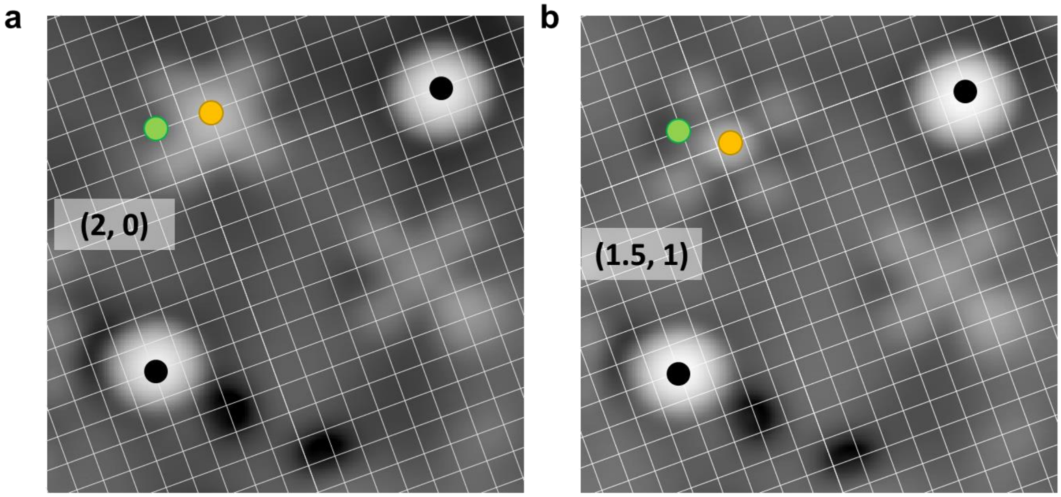

Supplementary Fig. 2. **Lattice site analysis of the Fe-FePc complex.** (a) States A and (b) B on MgO/Ag(001). The STM images (4 nm x 4 nm; V_{DC} = -100 mV, I = 20 pA) with the MgO lattice overlaid show that the lattice site difference between the FePc center (yellow) and the Fe adatom (green) for State A is (2, 0), and B is (1.5, 1), respectively. The other Fe adatoms (black; known to be adsorbed on oxygen sites) are labelled as the references.

Supplementary Note 3: Additional DFT calculations

In this section we elaborate the DFT results in greater detail.

3.1. Details on the energies of different FePc and complex configurations.

To rationalize the bistability and highlight the stabilizing role of the Fe atom on the complex, we systematically compared several configurations of FePc, both with and without an Fe adatom. Following our lattice analysis of the experimental results (Supplementary Fig. 2), we explored different alignments: some of them had the Fe atom of FePc on a top position, while in others it was in a bridge position (with respect to O atoms in MgO, see Supplementary Fig. 3). The summary of all differences in energy, charge transfer ΔN as well as magnetic moments are given in Supplementary Table 1. States A and B were identified as the lowest energy ones on top and bridge positions, respectively (Supplementary Fig. 4a). Here, the energy difference is 51 meV. In order to compare these results to pristine FePc in State A and State B (without Fe adatom) as done in Fig. 2d in the main text, we began from the State A and State B geometries with Fe adatom (Supplementary Fig. 4a), then removed the Fe adatom, and fully relaxed the system. The resulting energy difference between pristine FePc in State A and State B is 187 meV. The electronic configuration remains mostly identical (Supplementary Table 1). This comparison highlights the stabilizing role of the Fe adatom.

3.2. Spin Configuration of isolated FePc and complex A and B

In this section we discuss in greater detail the qualitative picture of the FePc spin states based on the DFT calculations and additionally comment on the isolated FePc spin state. Accurately modeling this system is challenging – not only due to the intrinsic complexity of FePc itself stemming from the multiorbital ground state [1-3] – but also because of its nontrivial interaction with the MgO/Ag(100) substrate: There is a significant charge transfer from the surface to the molecule, with FePc gaining approximately 1.5-2.0 electrons (see ΔN[FePc] in Supplementary Table 1. Supplementary Fig. 5 illustrates the most important configurations). This charge is transferred to the degenerate LUMO, which is mainly localized on the phthalocyanine (Pc) ligand. The electronic and magnetic structure of FePc is then dictated by how this additional charge is distributed. In State A, the charge rearrangement results in a nearly vanishing net magnetic moment on the Pc ligand (see μ[Pc] in Supplementary Table 1 and Supplementary Fig. 5c), yielding an overall spin state of approximately S ≈ 1, primarily localized on the Fe center (~2 μ_B). In contrast, in State B, the excess charge induces a Pc-centered magnetic moment of $(1.6-1.8)$ μ_B, antiferromagnetically coupled to the Fe spin, leading to a total spin state of S ≈ 0. This qualitative picture holds whether or not a nearby Fe adatom is present (Supplementary Fig. 4).

Thus, the overall spin state of FePc is primarily governed by the magnitude and organization of the surface-induced charge transfer.

We note, that the DFT results for FePc without a nearby Fe adatom (Supplementary Fig. 5b and Table 1) differ from those observed experimentally for isolated FePc, which exhibits a spin state of S = 1/2 [4] - not S = 1 as in the case of State A. We attribute this discrepancy between DFT and experiment to the system's sensitivity to charge transfer, which in turn is strongly dependent on the experimental conditions: In DFT, excess charge transfer from the MgO/Ag(001) substrate goes to the ligand (Supplementary Fig. 5b), leading to a vanishing ligand spin (-0.41μ_B). This is a larger value compared to the isolated molecule (Supplementary Fig. 5a) in gas phase, but still leads to an incorrect overall spin state (S~1). We tested that reducing ΔN restores a finite spin on the ligand and thus yields a correct result of a total spin (S=1/2). Therefore, in order to accurately describe pristine FePc, one not only has to correctly describe the molecule itself, but it is necessary to simultaneously account for the interaction with the surface, including the charge transfer. For example, combined experimental and theoretical studies have shown that the presence of interstitial oxygen between the MgO layers and Ag(001) significantly alters the charge transfer to a molecule by modifying the surface work function[5].

Importantly, the DFT calculations presented here effectively capture the qualitative electronic configurations associated with States A and B in the complex – a significant change in the ligand spin configuration – and how these lead to two bistable magnetic states for FePc.

3.3. Calculations of isolated Fe adatom

For the magnetic moment of the Fe adatom, we obtain S = 3/2 in both states A and B in the complex. However, for an isolated Fe adatom using the same computational parameters, we obtain S = 2 (μ = 3.90 μ_B), in agreement with the experimental observations [6]. When the Fe adatom is sufficiently far away (as in the solution H in Supplementary Fig. 3 and Table 1), a spin state of S=2 is obtained. Therefore, the change in the spin state is a result of the charge transfer of the adatom and its hybridization with FePc and the surface.

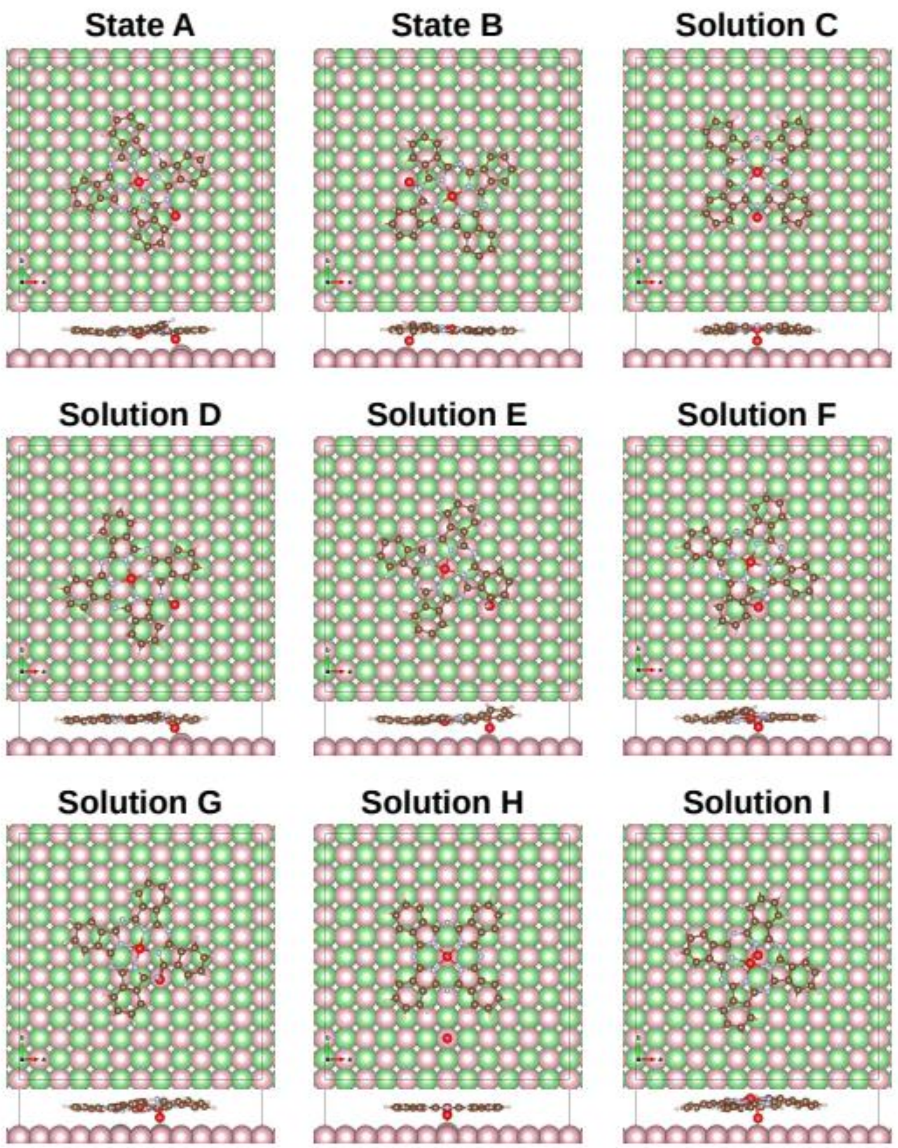

Supplementary Fig. 3. **DFT calculations of different FePc-Fe adsorption configurations.** Top view and side view images showing different configurations of the Fe-FePc complex on MgO/Ag(001) calculated by DFT. The first two configurations agree best in their orientation and lattice site with State A and State B observed in the experiment. The difference in binding energy between State A and State B is ~51 meV. Oxygen and magnesium atoms are indicated by pink and green spheres, respectively.

	ΔE (eV)	ΔN[FePc] (e-)	μ[FePc] (μ_B)	μ[Fe] (μ_B)	μ[Pc] (μ_B)	μ[Fe_{ad}] (μ_B)
**State A**	0.000	2.03	-2.08	-2.09	0.01	3.07
**State B**	0.051	1.86	0.16	1.84	-1.69	2.91
**Solution C**	0.134	1.82	0.11	1.84	-1.73	2.91
**Solution D**	0.233	1.87	-1.98	-2.05	0.07	2.97
**Solution E**	0.317	1.96	-0.12	-1.83	1.71	3.01
**Solution F**	0.458	2.00	-0.19	-1.83	1.64	3.09
**Solution G**	0.470	1.97	1.92	2.01	-0.09	2.99
**Solution H**	1.006	1.43	-1.28	-2.05	0.77	3.89
**Solution I**	1.011	1.69	0.15	1.79	-1.64	2.95

**Gas phase**	-	0.00	2.00	2.08	-0.08	-
**State A (w/o Fe_{ad})**	0.000	1.76	1.66	2.07	-0.41	-
**State B (w/o Fe_{ad})**	0.187	1.97	0.04	1.81	-1.77	-

Supplementary Table. 1. Corresponding energy differences from different solutions. Energy differences (ΔE) with respect to the lower energy configuration (State A), charge transfer of FePc (ΔN[FePc]) with respect to gas phase and magnetic moments for the different configurations shown in Supplementary Fig. 3. The magnetic moments are shown for FePc (μ[FePc]) and its components (Fe (μ[Fe]) and Pc (μ[Pc])), and for the Fe adatom (μ[Fe_{ad}]). Last two lines correspond to FePc without an extra Fe adatom in the configurations shown in supplementary Fig. 4. A positive magnetic moment denotes alignment with the fixed spin orientation of the Fe adatom, while a negative sign indicates antiparallel alignment.

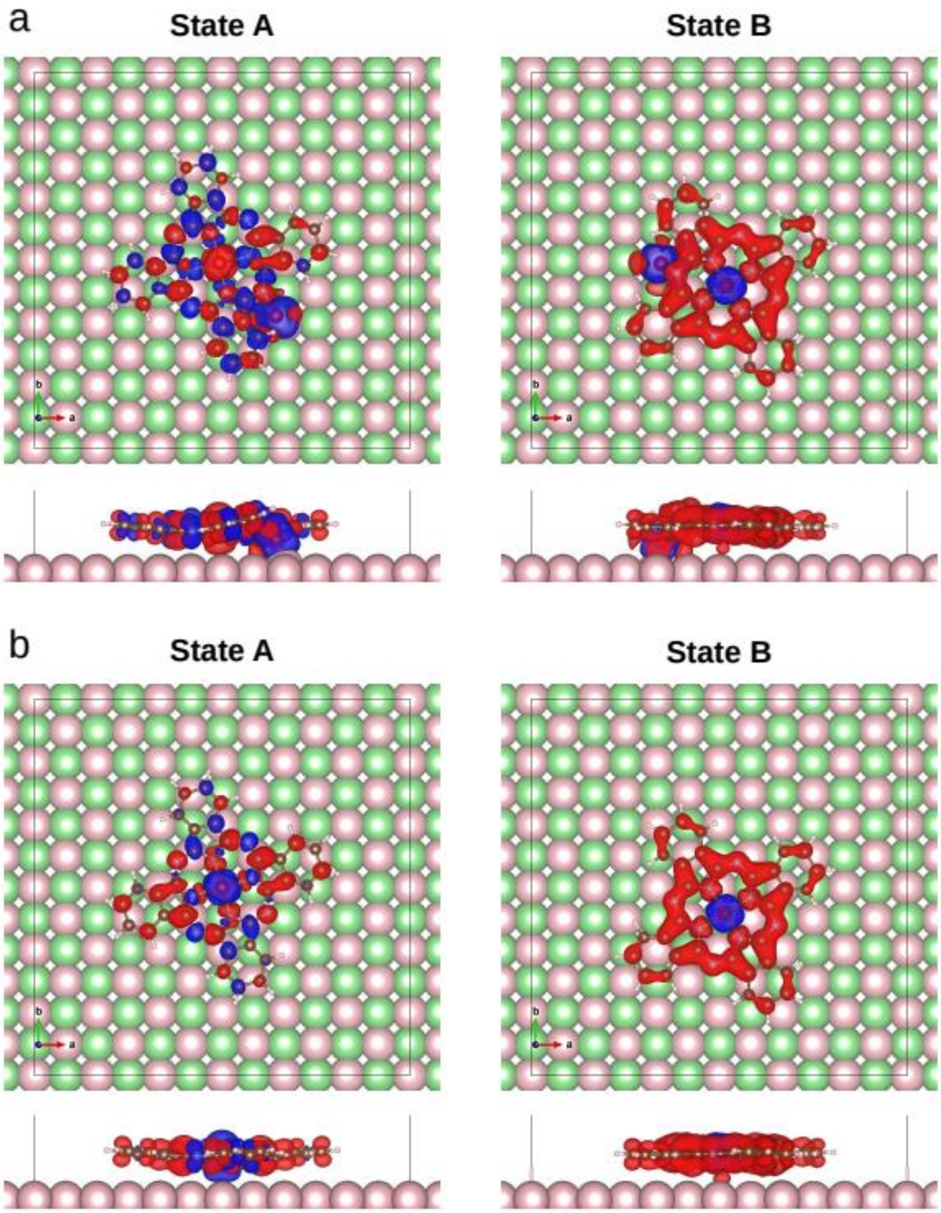

Supplementary Fig. 4. **DFT calculations of spin densities.** Top view and side view images showing the spin densities of States A and B (a) with Fe adatom and (b) without an extra Fe adatom. Blue (red) color represents majority (minority) spin.

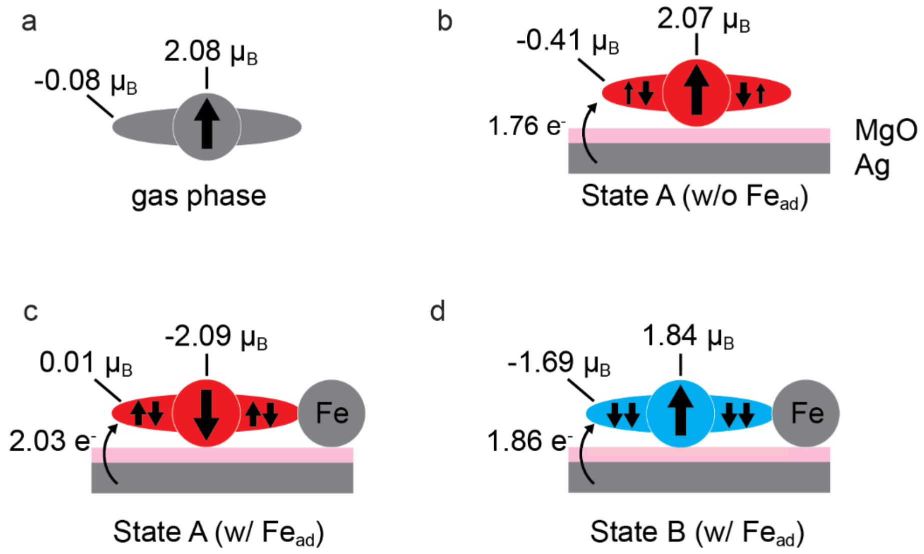

Supplementary Fig. 5. Schematic spin configurations of FePc in various conditions as obtained from DFT. (a) FePc in the gas phase. (b) Pristine FePc adsorbed on a 2 ML MgO/Ag(001) substrate without Fe adatom (State A). (c) FePc in State A with a nearby Fe adatom on 2 ML MgO/Ag(001). (d) FePc in State B with a nearby Fe adatom on 2 ML MgO/Ag(001). The magnetic moments of the central Fe atom (μ[Fe]) and the Pc ligand (μ[Pc])) are indicated along with the total charge transfer from the substrate to FePc (in units of electrons).

Supplementary Note 4: Reversible switching of State A and B

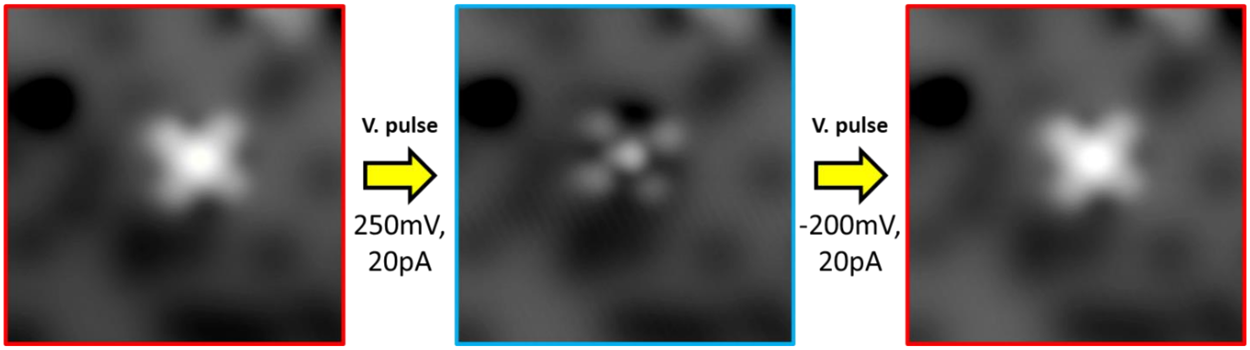

Supplementary Fig. 6. Reversible switching of States A and B. A sequence of STM voltage pulses induced switching between States A and B. Voltage pulse parameters: 250 mV, 20 pA for A→B; -200 mV, 20 pA for B→A. STM images (4 nm x 4 nm): State A: V_{DC} = -100 mV, I = 20 pA and State B: V_{DC} = 100 mV, I = 20 pA. The voltage pulses were applied above the molecule center.

Supplementary Note 5: Additional data of switching different Fe-FePc complexes

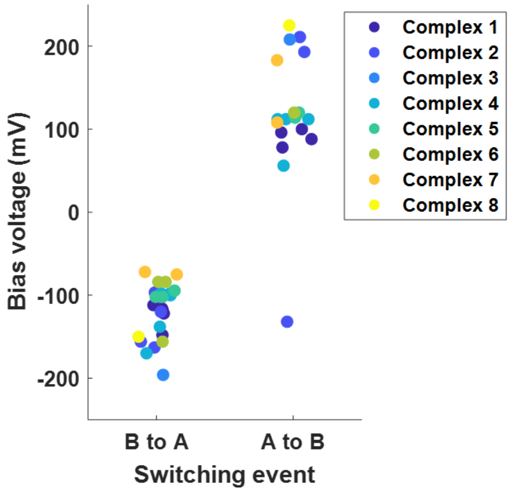

Supplementary Fig. 7. Switching of different complexes. Additional data for electric-field driven switching between States A and B on eight different complexes. One outlier (blue dot) on the wrong by switching from A to B was observed once.

Supplementary Note 6: Molecular rotation beyond the switching threshold

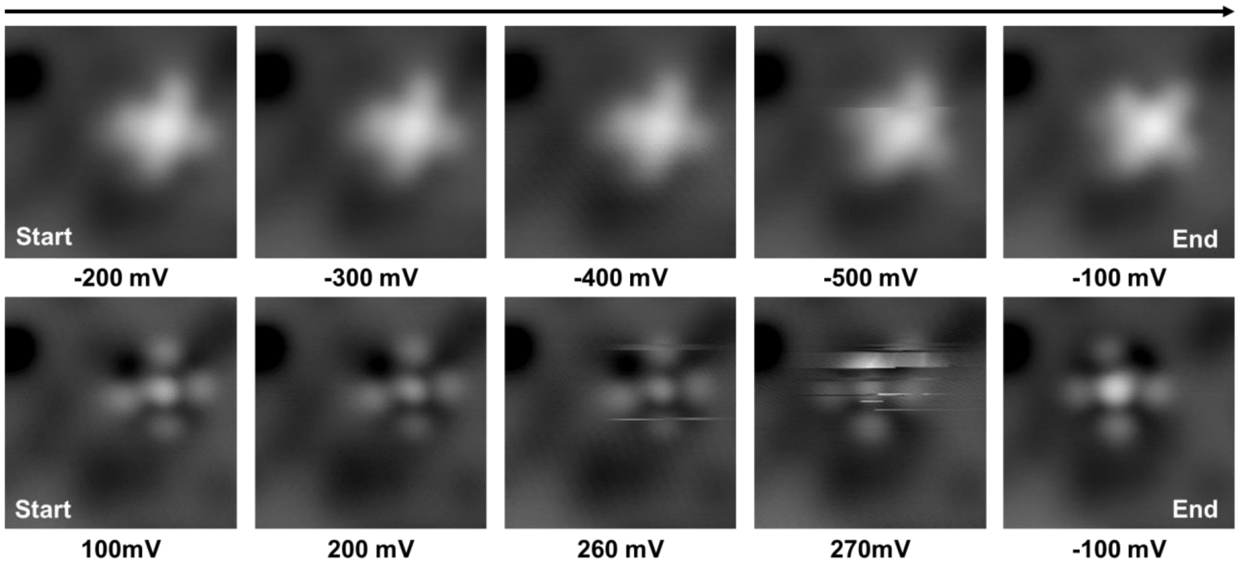

Supplementary Fig. 8. Molecular rotation beyond the switching threshold. STM images (3 nm x 3 nm) taken at different biases (I = 20 pA). After switching, increasing bias for STM scanning (from top to bottom) induces random motion of the Fe-FePc complexes without switching to the other states. Top row: State A. Bottom row: State B.

Supplementary Note 7: Additional statistical data for molecular rotation

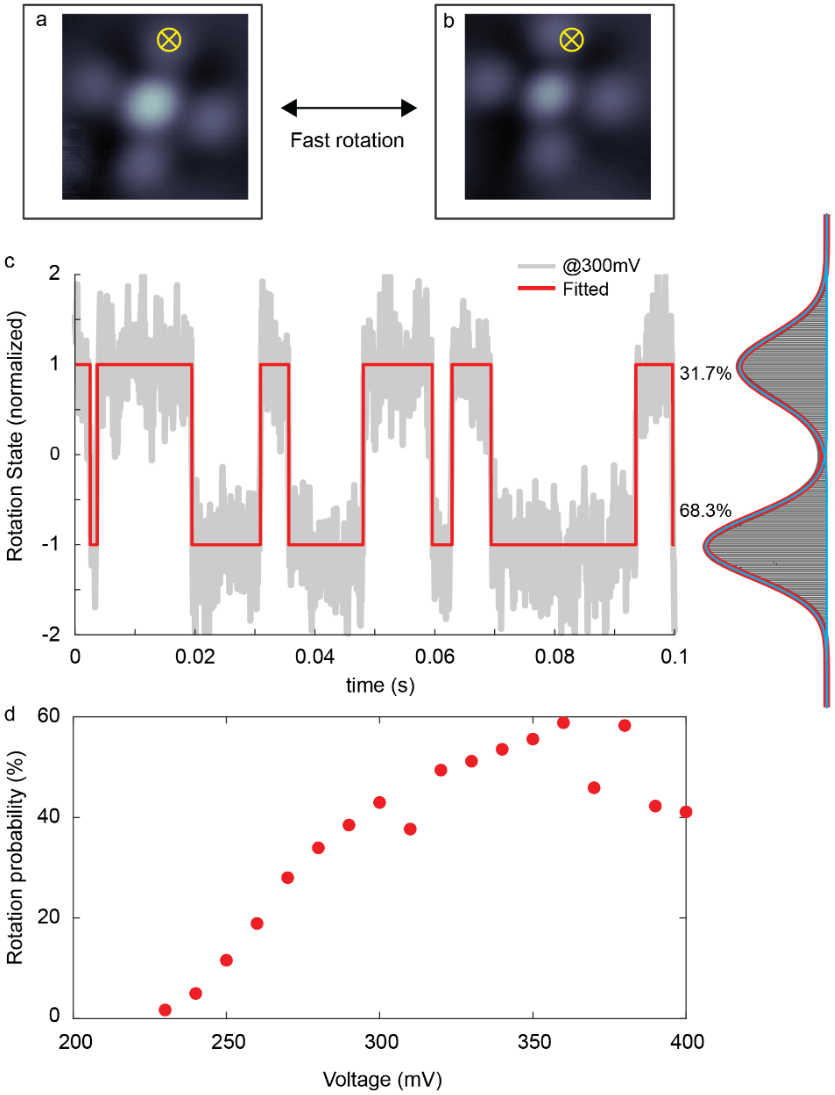

Supplementary Fig. 9. Molecular rotation at high bias voltage beyond the bistable region. STM images (1.5 nm x 1.5 nm) of the complex in State B: (a) before and (b) after rotation induced by applying a bias voltage of 300 mV. (c) By keeping the tip at a fixed position above the molecule (e.g., at the crossed circles in a and b) and disabling the feedback loop at V_{DC} = 300 mV, I = 20 pA, the orientation of the molecule can be monitored by tunneling current (gray). This setup statistically triggers switching back and forth between the two orientations. The current is normalized to -1 (lowest) and 1 (highest). Right: The histogram of the current distribution over a 60s timescale. (d) The rotation probability, defined by the occurrence of the highest current, increases with higher bias voltage.

Supplementary Note 8: Z-offset dependent I-V spectroscopy

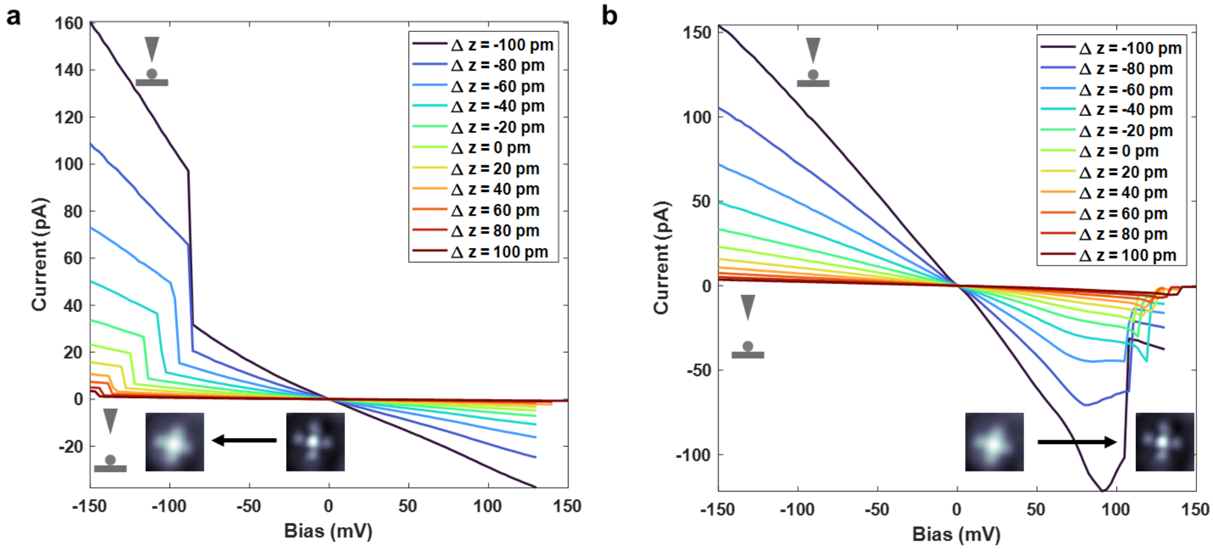

Supplementary Fig. 10. Z-offset dependent I(V) spectra. (a) The threshold voltage for switching from State B to A decreases as the tip moves closer to the molecule. (b) Similarly, the threshold voltage for switching from State A to B also decreases with reduced tip-sample distance. The feedback loop is opened at $\Delta z = 0$ (V_{DC} = -130 mV, I = 20 pA) and then moved according to the inset.

Supplementary Note 9: Setpoint dependent dI/dV spectra using a magnetic tip

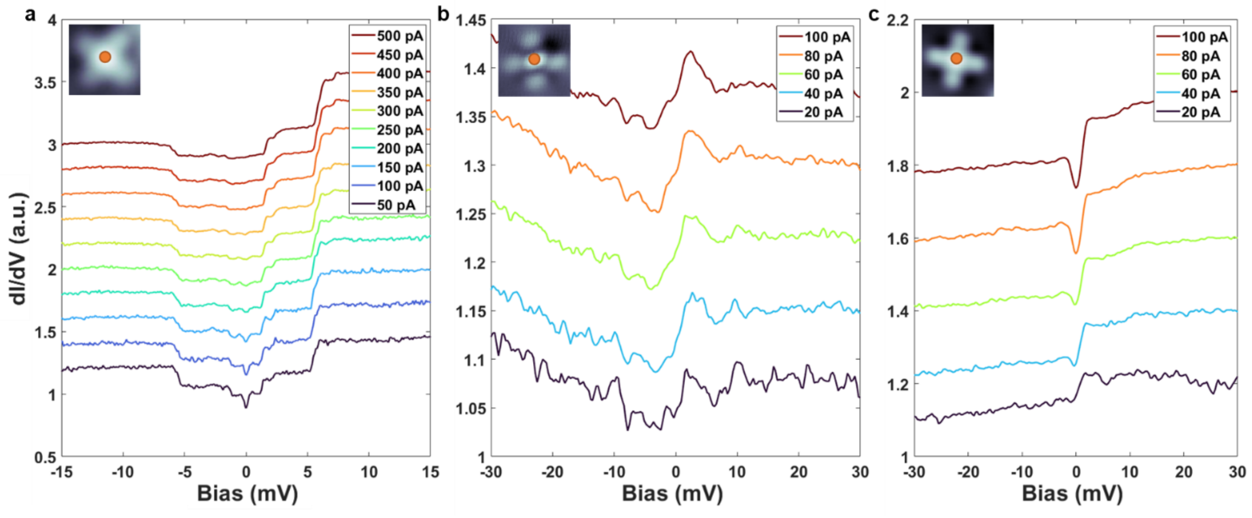

Supplementary Fig. 11. **Setpoint dependent dI/dV spectra using a magnetic tip.** Spin-polarized dI/dV spectra acquired at the center of FePc in State A (a), State B (b), and pristine FePc (c). Measurements for (a) were performed at zero magnetic field using tip #1, while those for (b) and (c) were conducted at a field of 0.5 T using tip #2. Bias voltages were set to $V_{DC} = -30$ mV for (a) and $V_{DC} = 30$ mV for (b) and (c). The pristine FePc exhibits an emerging zero-bias splitting in the dI/dV, characteristic of a spin S = 1/2. In contrast, FePc in State B shows no changes in the dI/dV with increasing setpoint, indicating a spin S = 0. In contrast, for FePc in State A, the levels additionally move in the presence of the tip magnetic field supporting its magnetic nature.

Supplementary Note 10: Spatially resolved dI/dV across the spin switch in State A

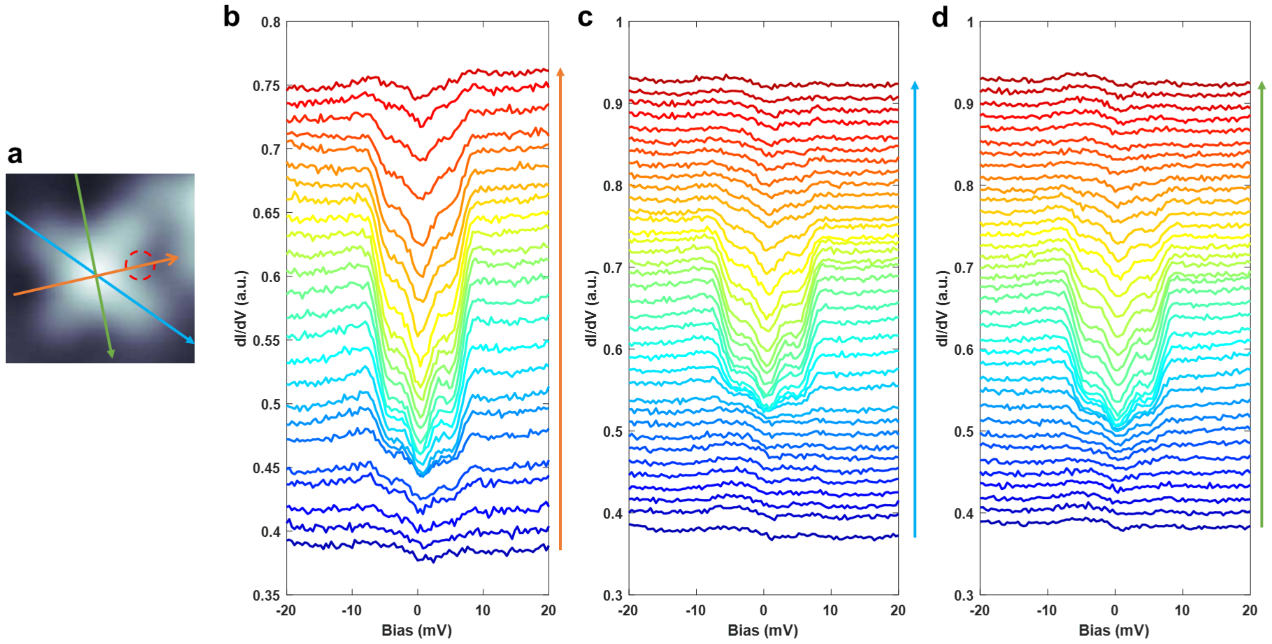

Supplementary Fig. 12. Spatially resolved dI/dV across the spin switch in State A. (a) The same topographic image as in Fig. 3b. (b-d) Spatially resolved dI/dV spectra along the colored arrows in (a) across the spin switch (-20 mV, 40 pA), acquired with relatively low energy resolution (101 data points). The dI/dV signal on the Fe site exhibits a similar feature to that at the FePc center but with a different step intensity.

Supplementary Note 11: Residual tip magnetic field effect

The g-factor of FePc on MgO/Ag(001) has been previously investigated and was found to be close to $g = 2$ [4]. For a spin S = ½ and g = 2, the expected resonance frequency is $f_0 = g\mu_B B/h = 13.55\ \mathrm{GHz}$ at 484 mT. However, the experimentally observed resonance frequency is ~12.8 GHz in Fig. 4 in the main text. This discrepancy arises from the additional magnetic field contribution of the spin-polarized tip [4], which we did not discuss in the main text for the sake of readability. Including the influence of B_{tip}, the resonance frequency is given by $hf_0 = g\mu_B(B_{ext} + B_{tip})$. In the given case, the magnetic tip is antiferromagnetically coupled to the molecular spin ($B_{tip} < 0$), which leads to a downward shift of approximately 0.75 GHz.

Supplementary Note 12: Resonance frequency shift of FePc target induced by spin switch

Fig. 4c in the main text shows the 53 MHz resonance frequency shift of the FePc target near to the bistable Fe-FePc switch. We attribute that shift to the change in magnetic properties between the two states. Similarly, changing the direction of the magnetic state in rare-earth adatoms on MgO resulted in a frequency shift [7, 8]. However, often when two spins are coupled in ESR-STM experiments, a splitting of the resonance and two emergent peaks are measured [4, 9], that correspond to the two configurations of the remote spin's ground state doublet. Their relative intensity is then given by the time average of its thermal occupation. In this experiment we only observe one peak that is shifted for both states. For State B a likely trivial answer is that it is non-magnetic, as suggested by the dI/dV measurements, and thus does not lead to a splitting. For State A, however, one could expect multiple peaks as it is the case for e.g. dimers of FePc molecules [4]. Thus, this indicates that either the spin state is very long lived – as it is the case for rare-earth ions [7, 8] and that it is stable over the timescale of the experiment. Alternatively, the magnetic moment is large enough that the peak of the excited state is "frozen out", so that it is not thermally populated and thus is not visible. We believe the latter to be the case here, since a long-lived state has not been observed for the constituents - FePc or Fe atoms - and is only obtained under very particular conditions. To estimate the visibility of a second state, we calculate the spins states and eigenenergies of the system using the Hamiltonian in Eq. (1) in the main text. The corresponding energy diagram is shown for State A in Supplementary Fig. 13. The switch in State A at 0.5 T exhibits a ferromagnetic ground state where both spins align. This state has the highest population at low temperatures (100% at 50 mK [cryostat temperature]; 90% at 300 mK [highest temperature estimate during ESR experiments, which can heat up the junction]). Thus, the populations of any state other than the ground state is very low and the intensity of a corresponding ESR peak will be vanishingly small. This effect will be enhanced further by including an orbital moment for FePc, which increases the g-factor. In different experimental [10-12] and theoretical [2, 3] works, values of up to g = 2.4 were found for FePc, which increases the ground state population (100% at 50 mK; 93% at 300 mK) even further.

Next, we discuss the magnitude and direction of the shift: Using the formula for dipolar coupling: $hf_{dipolar} = \frac{\mu_0}{2\pi}\frac{1}{r^3} m_z^{FePc} \cdot m_z^{switch}$ and a distance of $r = 1.68$ nm (from Supplementary Fig. 14a), we calculate a dipolar coupling of ~22 MHz between FePc target (S = 1/2) and switch (S = 1 for molecule) in State A (We here neglect the influence of the Fe adatom, since it stays constant ~89 MHz in both configurations). However, this dipolar interaction would shift the resonance frequency of the FePc target spin towards *lower* frequencies relative to that in case of State B. A possible mechanism that explains the shift is the dominance of exchange interaction as it has been observed also in FePc-FePc dimers over this length scale [4]. Consequently, the Δf can be rationalized by an additional exchange interaction of 22 MHz $+ 53$ MHz $= 75$MHz. For that, the exchange coupling between the target FePc and the spin switch must be ferromagnetic. This positive contribution would compensate for the dipolar coupling, resulting in a net upward shift of the resonance frequency. Normally, antiferromagnetic exchange is observed for on-surface spins [4, 9]. One possibility is that the residual spin of the Pc ligand plays a more important role in the spin

coupling, which is not accounted for in our simple spin Hamiltonian in the main text. Thus, it would play the role of a mediator that leads to an effective ferromagnetic coupling between the spins.

Supplementary Note 13: Energy level diagram of the State A complex

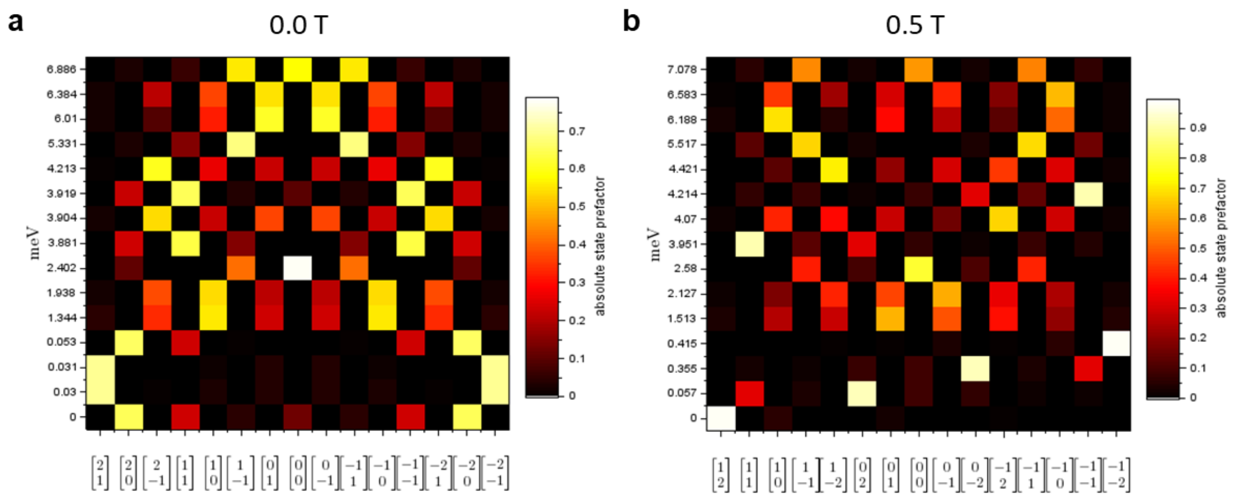

Supplementary Fig. 13. Heatmaps showing the energy level diagram of the complex in State A under (a) 0 T and (b) 0.5 T magnetic fields. In State A, an Fe spin S = 2 (D = -0.86 meV, E = 0.11 meV, g = 2.6 [13] is coupled to an FePc S = 1 (D = 1.3 meV, g = 2.0), and the coupling strength is J = -0.88 meV (ferromagnetic). The color scale represents the absolute state prefactor, with brighter colors corresponding to higher values. The diagram is calculated according to ref. [14]. The thermal population of the ground state is estimated using $p_0 = \frac{1}{1+\exp\left(-\frac{\Delta E}{k_B T}\right)}$, where ΔE is the energy difference between the excited and ground states, k_B is the Boltzmann constant and T is the temperature.

Supplementary Note 14: Lattice fit analysis of State A and B

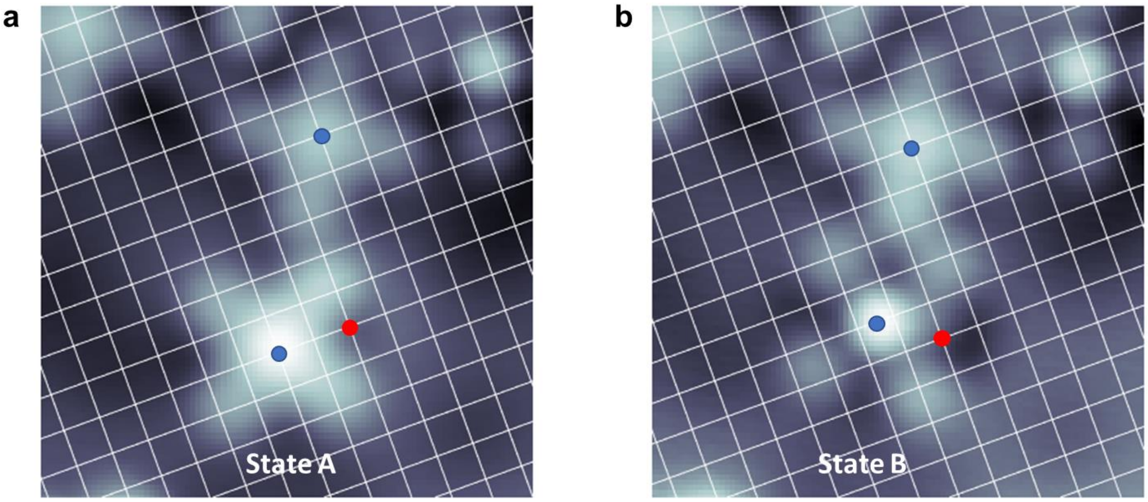

Supplementary Fig. 14. Lattice fit of the structure shown in Fig. 4 in the main text: a target FePc molecule coupled to an Fe-FePc spin switch in State A (a) and State B (b). In State A, the lattice site distance between the molecule centers corresponds to (3, 5) MgO lattice sites, while in State B, it is (2.5, 4). The Fe atom location remained unchanged.

References

1. Mugarza, A. et al. Electronic and magnetic properties of molecule-metal interfaces: Transition-metal phthalocyanines adsorbed on Ag(100). *Physical Review B* **85**, 155437 (2012).
2. Natoli, C.R., Krüger, P., Bartolomé, J. & Bartolomé, F. Determination of the ground state of an Au-supported FePc film based on the interpretation of Fe K- and L-edge x-ray magnetic circular dichroism measurements. *Physical Review B* **97**, 155139 (2018).
3. Natoli, C.R., Krüger, P., Yoshimoto, Y., Bartolomé, J. & Bartolomé, F. Simple model of the ground state and spin-orbital excitations of free and adsorbed Fe(II) phthalocyanine molecules. *Physical Review B* **98**, 195108 (2018).
4. Zhang, X. et al. Electron spin resonance of single iron phthalocyanine molecules and role of their non-localized spins in magnetic interactions. *Nature Chemistry* **14**, 59-65 (2022).
5. Pal, J. et al. How Growing Conditions and Interfacial Oxygen Affect the Final Morphology of MgO/Ag(100) Films. *The Journal of Physical Chemistry C* **118**, 26091-26102 (2014).
6. Baumann, S. et al. Origin of Perpendicular Magnetic Anisotropy and Large Orbital Moment in Fe Atoms on MgO. *Physical Review Letters* **115**, 237202 (2015).
7. Reale, S. et al. Electrically driven spin resonance of 4f electrons in a single atom on a surface. *Nature Communications* **15**, 5289 (2024).
8. Singha, A. et al. Engineering atomic-scale magnetic fields by dysprosium single atom magnets. *Nature Communications* **12**, 4179 (2021).
9. Choi, T. et al. Atomic-scale sensing of the magnetic dipolar field from single atoms. *Nature Nanotechnology* **12**, 420-424 (2017).
10. Tsukahara, N. et al. Adsorption-Induced Switching of Magnetic Anisotropy in a Single Iron(II) Phthalocyanine Molecule on an Oxidized Cu(110) Surface. *Physical Review Letters* **102**, 167203 (2009).

11. Barraclough, C.G., Martin, R.L., Mitra, S. & Sherwood, R.C. Paramagnetic Anisotropy, Low Temperature Magnetization, and Electronic Structure of Iron(II) Phthalocyanine. *The Journal of Chemical Physics* **53**, 1643-1648 (1970).
12. Bartolomé, J. et al. Highly unquenched orbital moment in textured Fe-phthalocyanine thin films. *Physical Review B* **81**, 195405 (2010).
13. Paul, W. et al. Control of the millisecond spin lifetime of an electrically probed atom. *Nature Physics* **13**, 403-407 (2017).
14. Ternes, M. Spin excitations and correlations in scanning tunneling spectroscopy. *New Journal of Physics* **17**, 063016 (2015).
