# Supplementary figures and images for: An electrically controlled single-molecule spin switch
Source: Nat Commun. 2025 Sep 8;16:8242. doi: 10.1038/s41467-025-63574-0 (PMC12417546; doi:10.1038/s41467-025-63574-0)

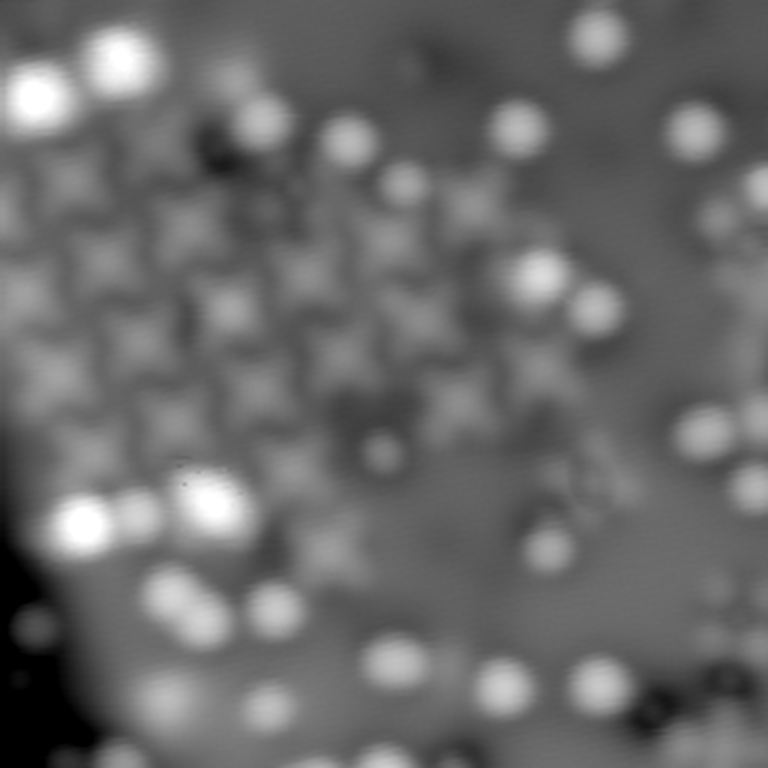

Supplement: Supplementary file 3 — Source Data [file 41467_2025_63574_MOESM3_ESM.zip › SourceData/Figure1a/Topo_Ag_202304128_10nm.png]

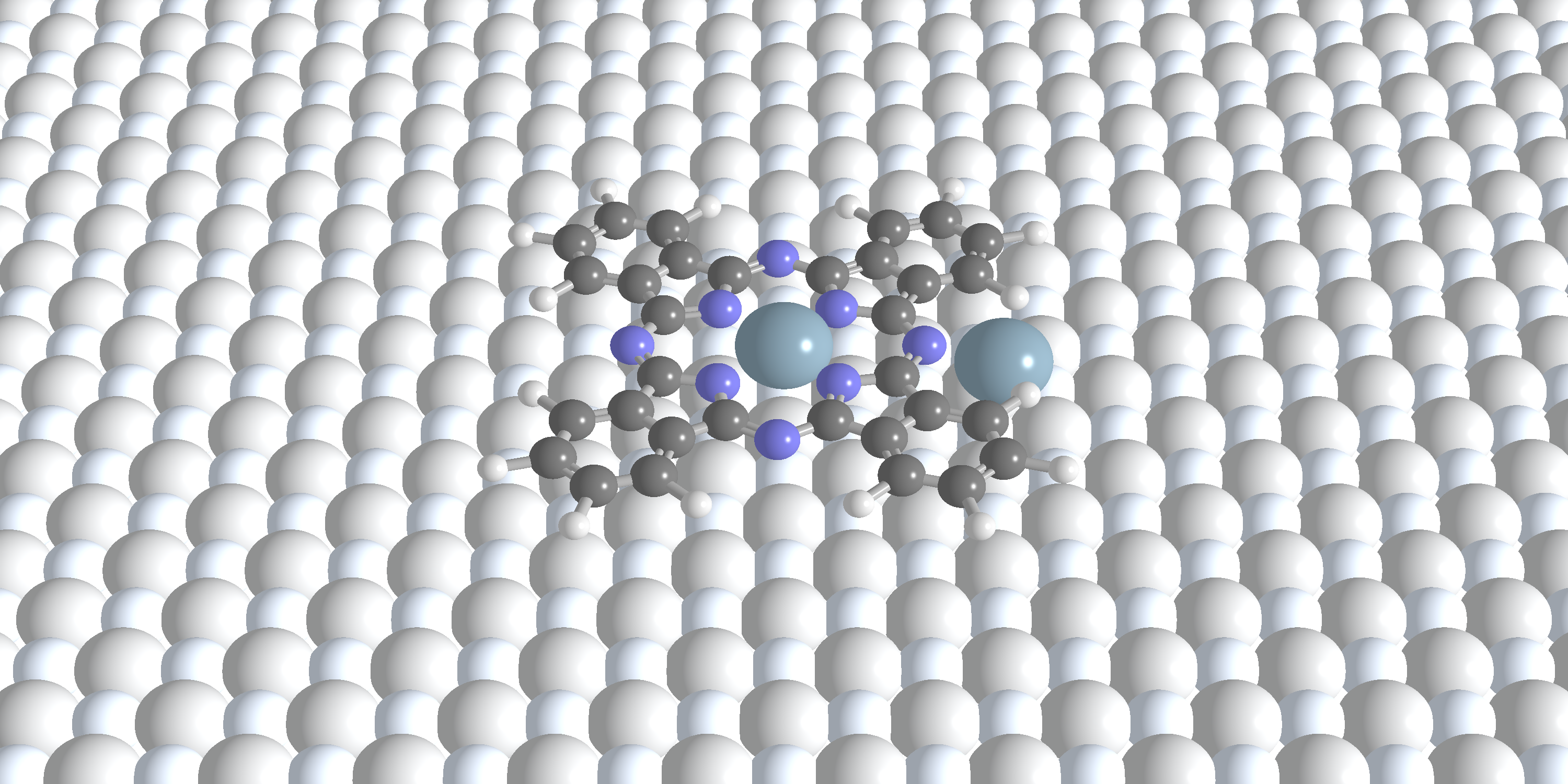

Supplement: Supplementary file 3 — Source Data [file 41467_2025_63574_MOESM3_ESM.zip › SourceData/figure1bcd/Povray/SwitchyDimer.png]

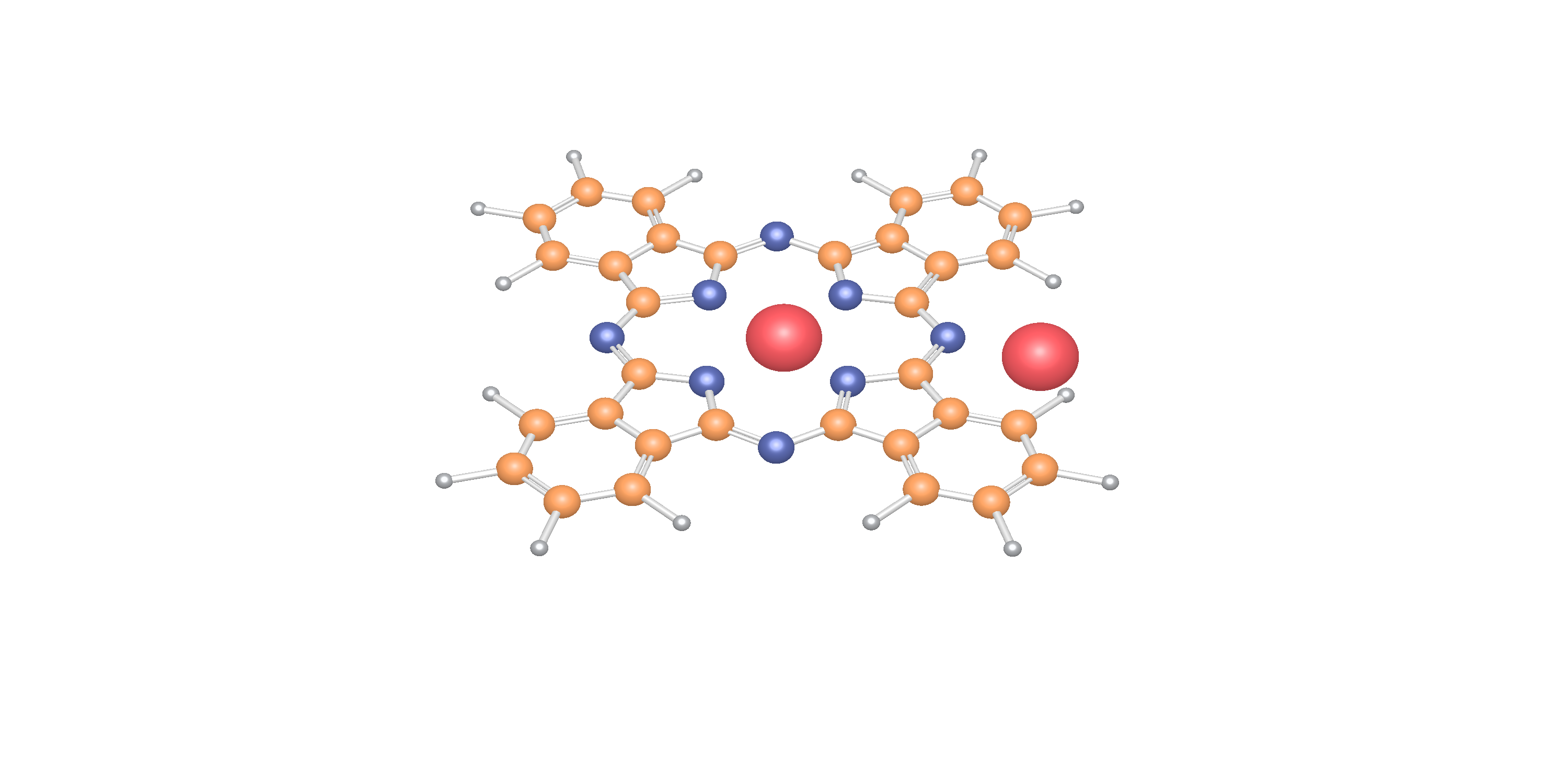

Supplement: Supplementary file 3 — Source Data [file 41467_2025_63574_MOESM3_ESM.zip › SourceData/figure1bcd/Povray/SwitchyDimerNoTipNoSurface.png]

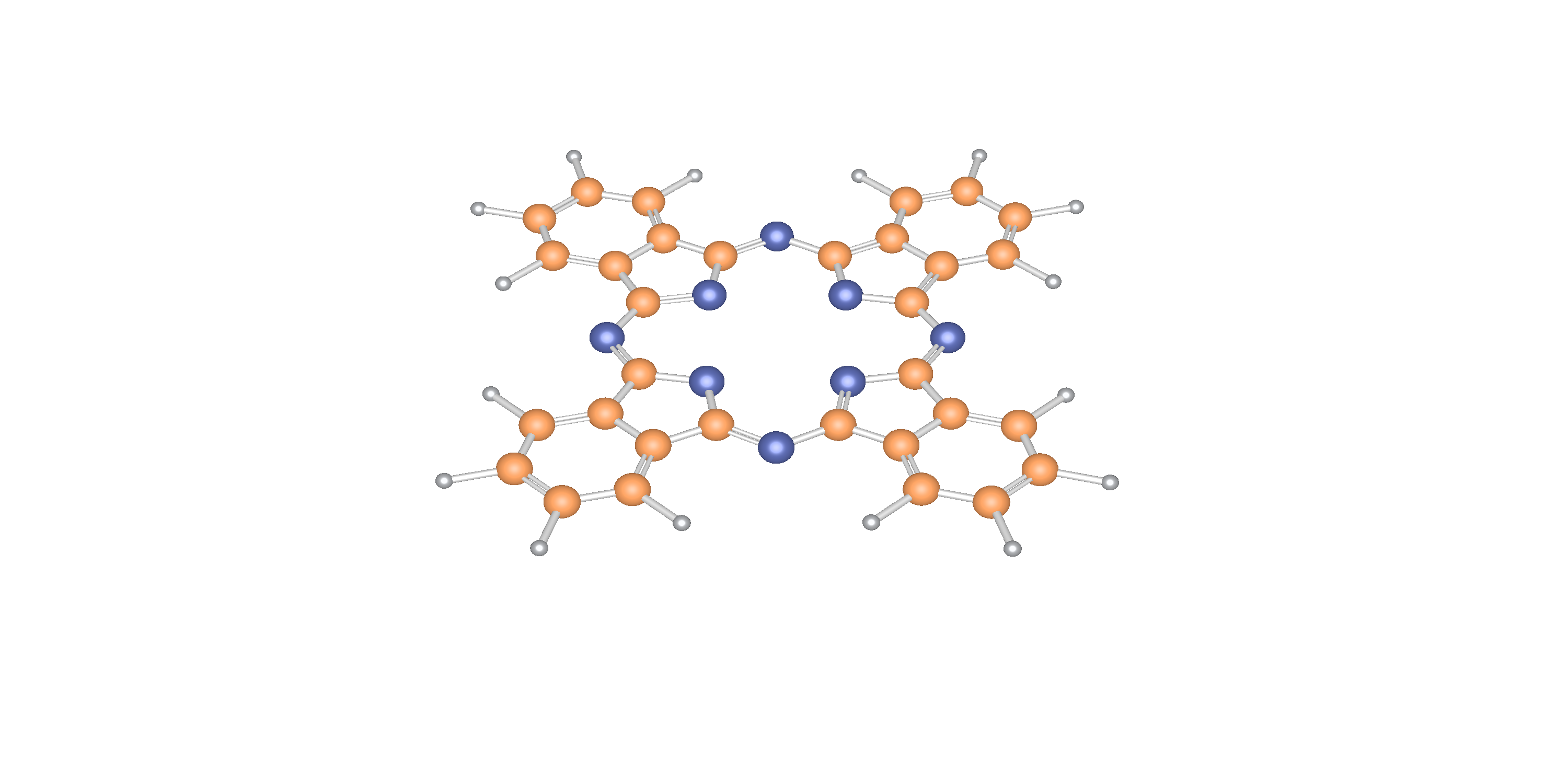

Supplement: Supplementary file 3 — Source Data [file 41467_2025_63574_MOESM3_ESM.zip › SourceData/figure1bcd/Povray/SwitchyDimerNoTipNoSurfaceNoFe.png]

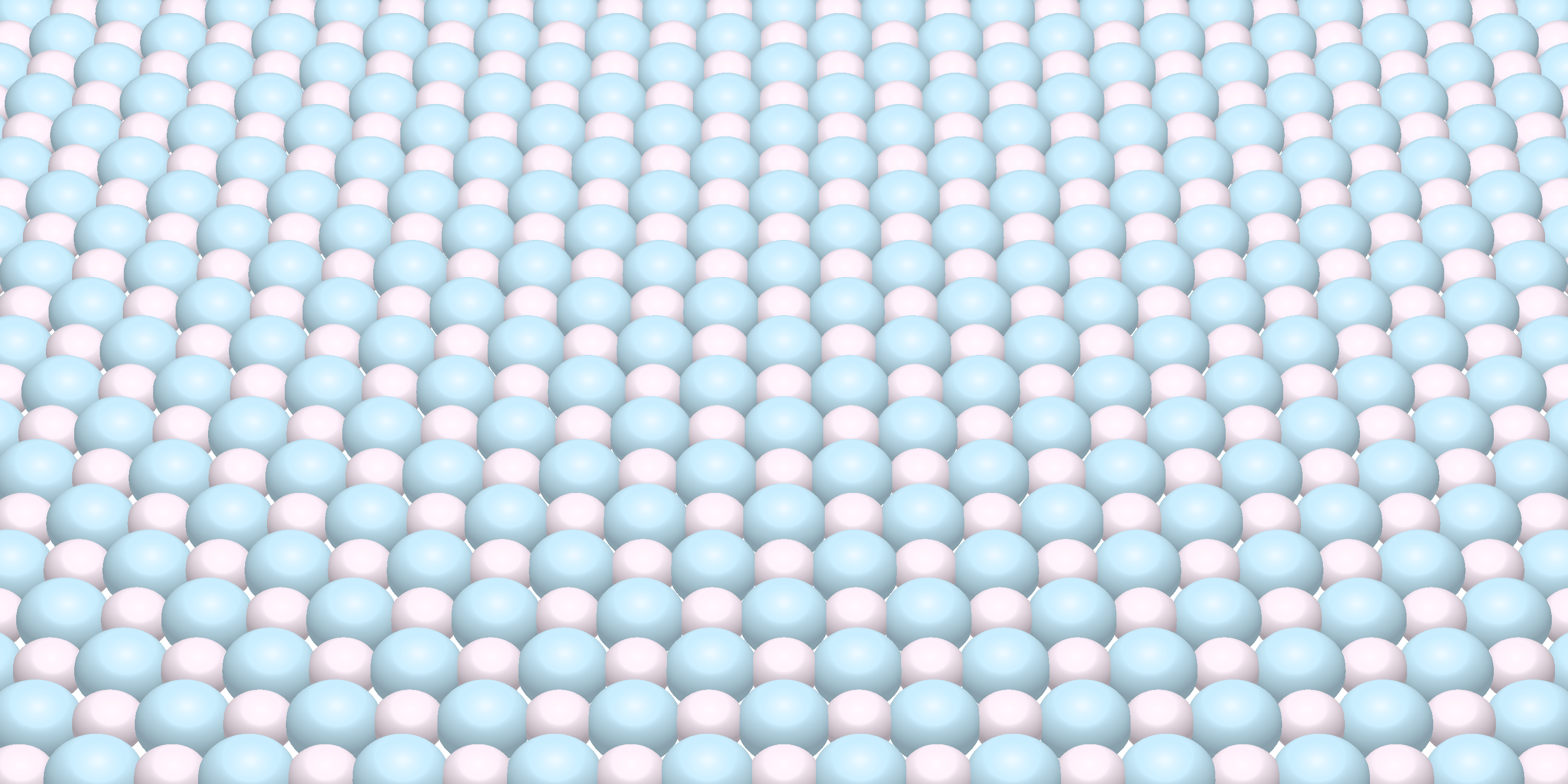

Supplement: Supplementary file 3 — Source Data [file 41467_2025_63574_MOESM3_ESM.zip › SourceData/figure1bcd/Povray/SwitchyDimer_NoMolecule.png]

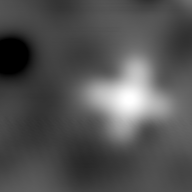

Supplement: Supplementary file 3 — Source Data [file 41467_2025_63574_MOESM3_ESM.zip › SourceData/figure1bcd/Topo_switch/Ag100_MgO_Fe_FePc_June1929.png]

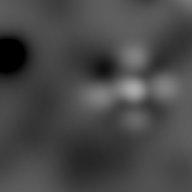

Supplement: Supplementary file 3 — Source Data [file 41467_2025_63574_MOESM3_ESM.zip › SourceData/figure1bcd/Topo_switch/Ag100_MgO_Fe_FePc_June1930.png]

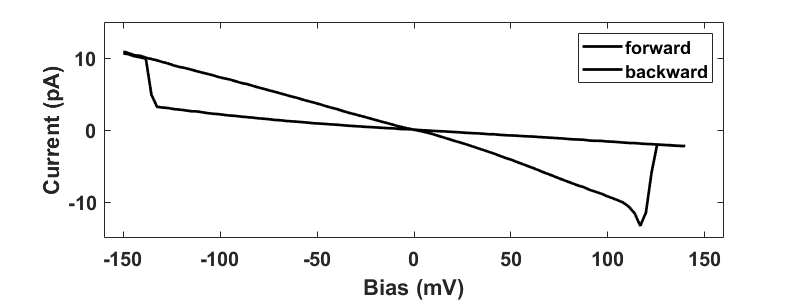

Supplement: Supplementary file 3 — Source Data [file 41467_2025_63574_MOESM3_ESM.zip › SourceData/Figure2a/IV.tif]

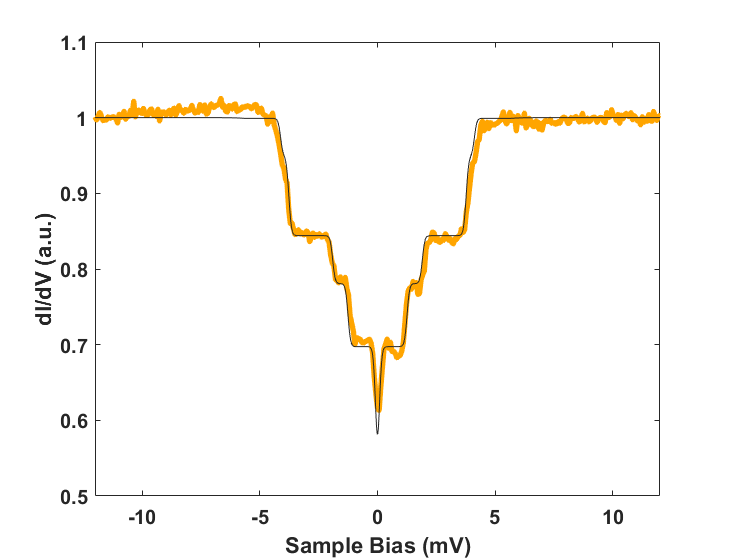

Supplement: Supplementary file 3 — Source Data [file 41467_2025_63574_MOESM3_ESM.zip › SourceData/figure3a/excellentFit.tif]

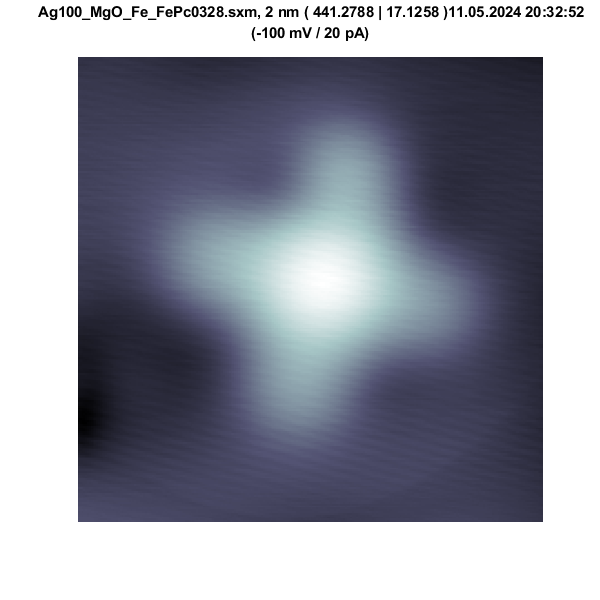

Supplement: Supplementary file 3 — Source Data [file 41467_2025_63574_MOESM3_ESM.zip › SourceData/Figure3bcef/brightTopo.tif]

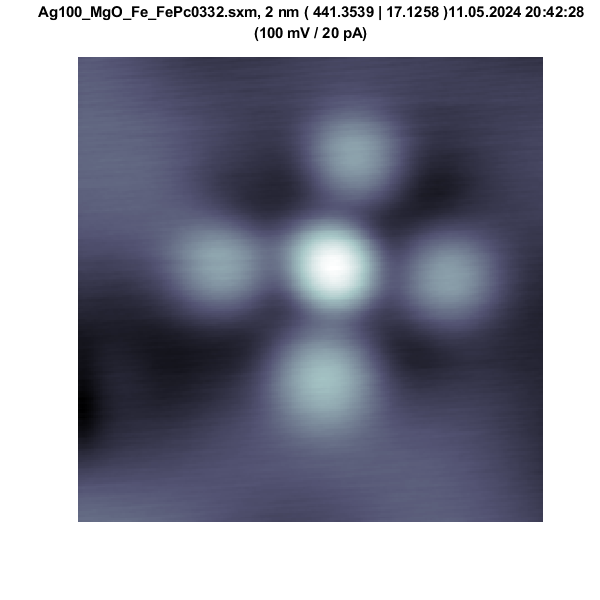

Supplement: Supplementary file 3 — Source Data [file 41467_2025_63574_MOESM3_ESM.zip › SourceData/Figure3bcef/darkTopo.tif]

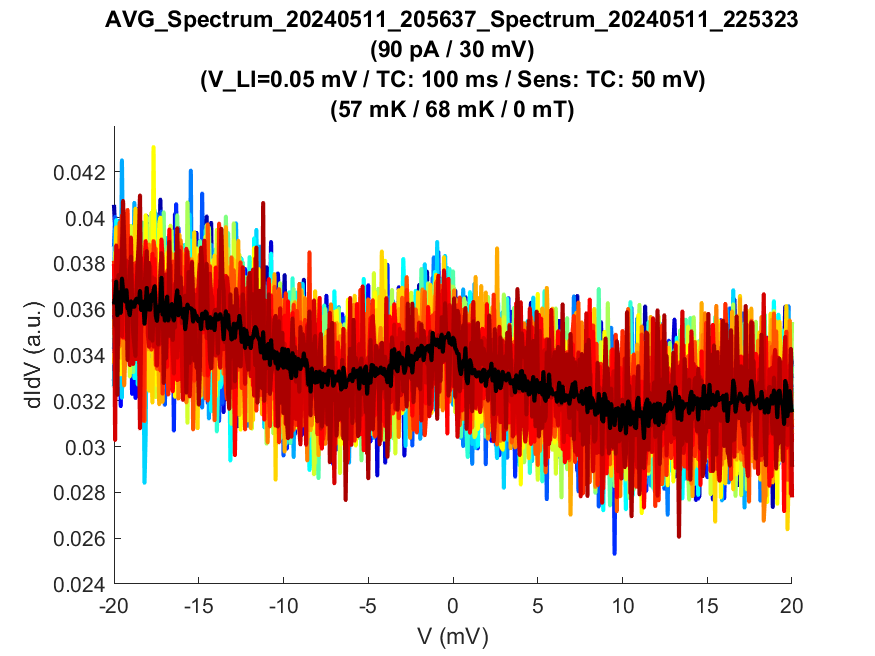

Supplement: Supplementary file 3 — Source Data [file 41467_2025_63574_MOESM3_ESM.zip › SourceData/figure3d/AVG_Spectrum_20240511_205637_Spectrum_20240511_225323.png]

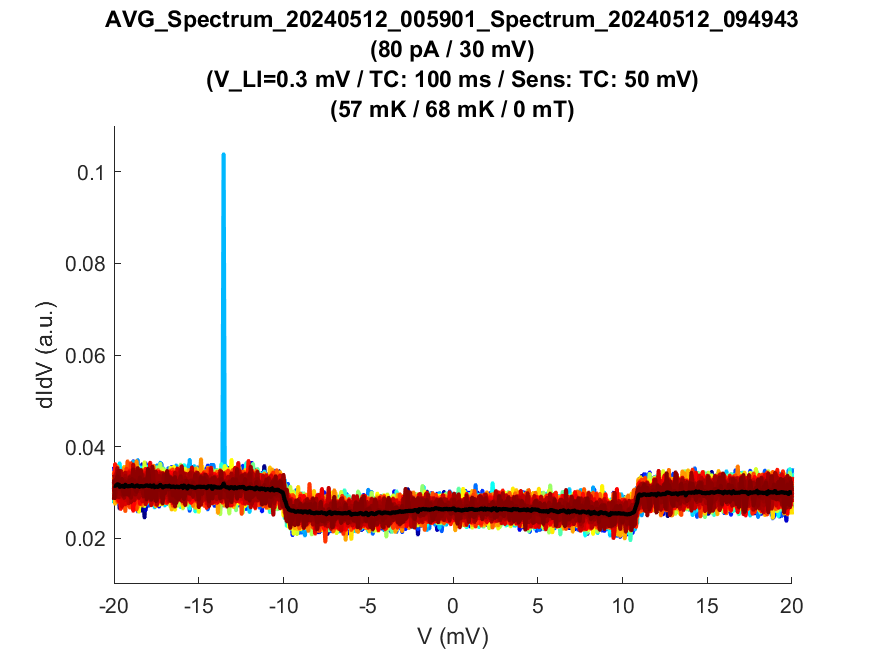

Supplement: Supplementary file 3 — Source Data [file 41467_2025_63574_MOESM3_ESM.zip › SourceData/figure3d/AVG_Spectrum_20240512_005901_Spectrum_20240512_094943.png]

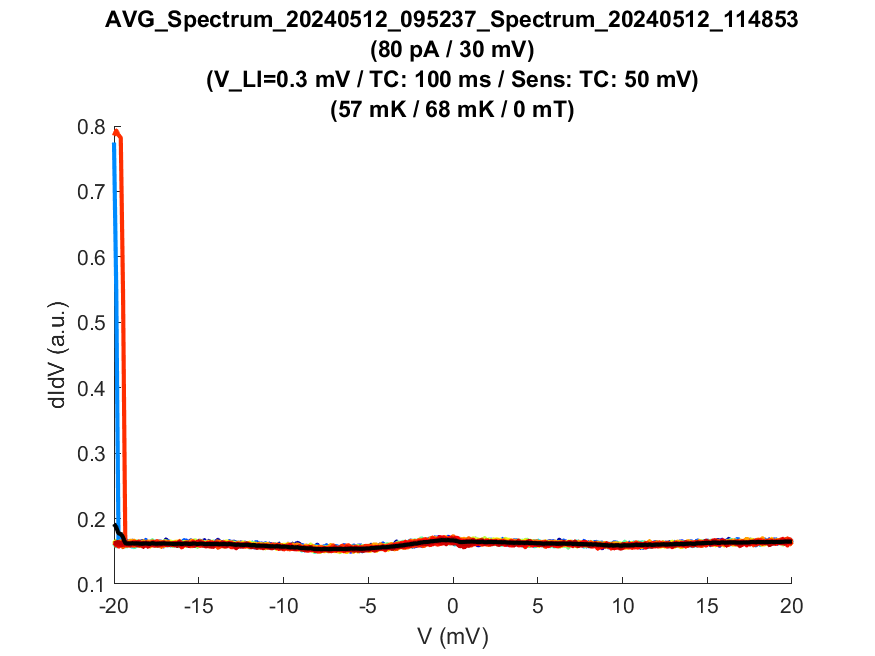

Supplement: Supplementary file 3 — Source Data [file 41467_2025_63574_MOESM3_ESM.zip › SourceData/figure3d/AVG_Spectrum_20240512_095237_Spectrum_20240512_114853.png]

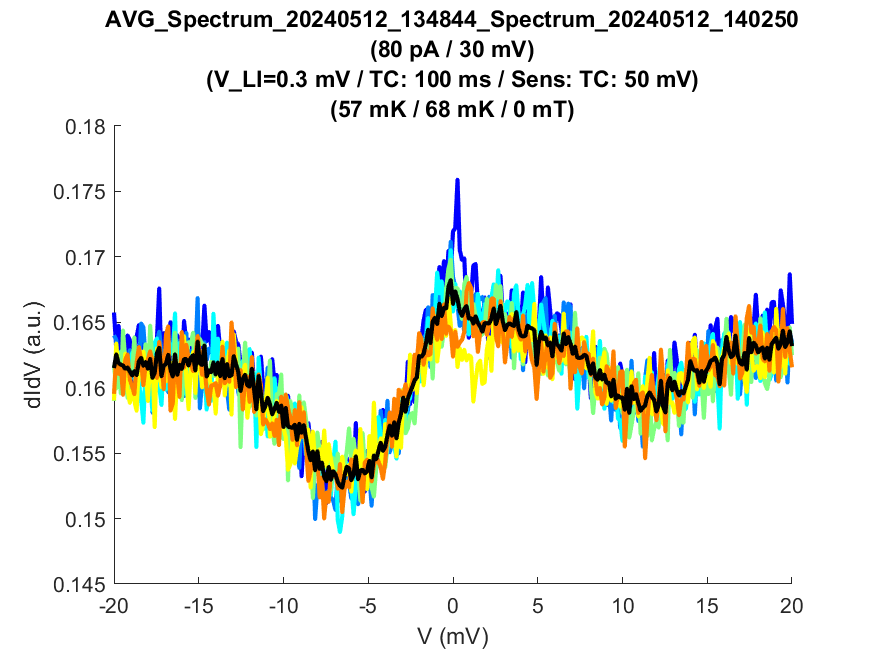

Supplement: Supplementary file 3 — Source Data [file 41467_2025_63574_MOESM3_ESM.zip › SourceData/figure3d/AVG_Spectrum_20240512_134844_Spectrum_20240512_140250.png]

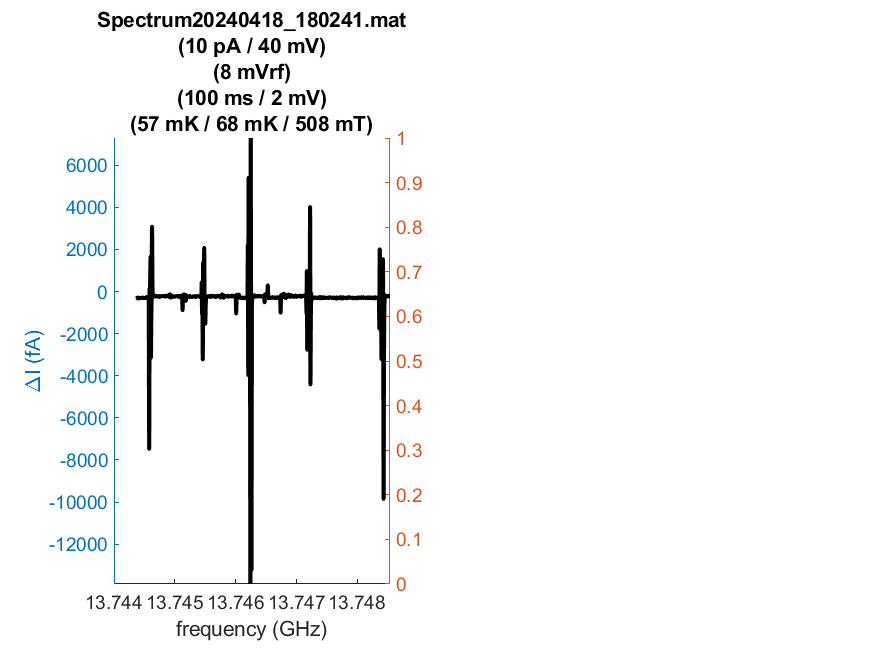

Supplement: Supplementary file 3 — Source Data [file 41467_2025_63574_MOESM3_ESM.zip › SourceData/figure4/ESR_resonance_OnOff_switch/Spectrum20240418_180241.png]

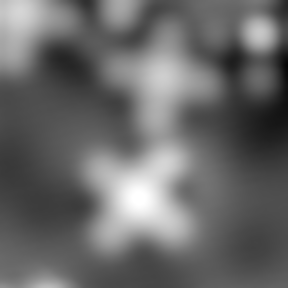

Supplement: Supplementary file 3 — Source Data [file 41467_2025_63574_MOESM3_ESM.zip › SourceData/figure4/Topo_CoupledWithFePc/Ag100_MgO_Fe_FePc2047.png]

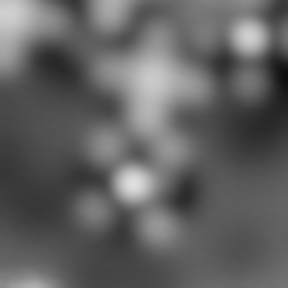

Supplement: Supplementary file 3 — Source Data [file 41467_2025_63574_MOESM3_ESM.zip › SourceData/figure4/Topo_CoupledWithFePc/Ag100_MgO_Fe_FePc2049.png]
